# The Study of Alternative Splicing Events in Human Induced Pluripotent Stem Cells From a Down's Syndrome Patient

**DOI:** 10.3389/fcell.2021.661381

**Published:** 2021-09-30

**Authors:** Yunjie Wang, Zexu Li, Guanheng Yang, Linlin Cai, Fan Yang, Yaqiong Zhang, Yitao Zeng, Qingwen Ma, Fanyi Zeng

**Affiliations:** ^1^Shanghai Institute of Medical Genetics, Shanghai Children's Hospital, Shanghai Jiao Tong University, Shanghai, China; ^2^National Health Commission Key Laboratory of Embryo Molecular Biology, Shanghai Key Laboratory of Embryo and Reproduction Engineering, Shanghai, China; ^3^Department of Histoembryology, Genetics & Development, Shanghai Jiao Tong University School of Medicine, Shanghai, China

**Keywords:** Down's syndrome, alternative splicing, iPSCs, gene regulation, differentially expressed genes

## Abstract

Down's syndrome (DS) is one of the most commonly known disorders with multiple congenital disabilities. Besides severe cognitive impairment and intellectual disability, individuals with DS also exhibit additional phenotypes of variable penetrance and severity, with one or more comorbid conditions, including Alzheimer's disease, congenital heart disease, or leukemia. Various vital genes and regulatory networks had been studied to reveal the pathogenesis of the disease. Nevertheless, very few studies have examined alternative splicing. Alternative splicing (AS) is a regulatory mechanism of gene expression when making one multi-exon protein-coding gene produce more than one unique mature mRNA. We employed the GeneChip Human Transcriptome Array 2.0 (HTA 2.0) for the global gene analysis with hiPSCs from DS and healthy individuals. Examining differentially expressed genes (DEGs) in these groups and focusing on specific transcripts with AS, 466 up-regulated and 722 down-regulated genes with AS events were identified. These genes were significantly enriched in biological processes, such as cell adhesion, cardiac muscle contraction, and immune response, through gene ontology (GO) analysis of DEGs. Candidate genes, such as *FN1* were further explored for potentially playing a key role in DS. This study provides important insights into the potential role that AS plays in DS.

## 1. Introduction

Down's syndrome (DS) or trisomy 21 (OMIM #190685) is a well-recognized and studied complex genetic condition caused by a chromosomal disorder, namely the presence of a total or partial trisomy of chromosome 21 (HSA21). It occurs in ~1:700–1:1,000 newborns globally (Weijerman and de Winter, 2010), and it is the most commonly known genetic etiology associated with moderate to severe intellectual disability. Furthermore, individuals with DS also exhibit additional phenotypes of variable penetrance and severity, with one or more comorbid conditions, including Alzheimer's disease, congenital heart disease, or leukemia (Ballard et al., 2016). Further understanding of the relationship between redundant chromosome 21 and its associated diseases is expected to provide theoretical support for revealing the pathogenesis, and developing therapeutic approaches and drugs to treat DS (Ballard et al., 2016). Moreover, due to the supernumerary copy of chromosome 21 (HSA21), some researchers hypothesized that the most dosage-sensitive genes in chromosome 21 are likely to contribute to the DS phenotype, also known as the “gene dosage effect” hypothesis (Korenberg et al., 1994). Some results support the “gene dosage effect” hypothesis that most of the Down's syndrome phenotypes are related to alterations in gene expression due to the extra chromosome 21 (HSA21). However, some researchers doubted that the DS phenotype could merely be explained by gene dosage effects (Jiang et al., 2013), the hypothesis of favor among medical geneticists. The factors influencing DS phenotypes include chromosome 21 DNA, functional elements and variability of chromosome 21, the variability of other chromosomes, chromatin structure, epigenetic modifications, stochastic events, and the environment (Antonarakis, 2017).

Nevertheless, very few studies on DS to date have examined alternative splicing (AS), a widespread regulatory mechanism of gene expression and makes one multi-exon protein-coding gene capable of producing more than one unique mature mRNA. AS provides transcriptional plasticity by controlling which RNA isoforms are expressed at a given time point in a given cell type. Alternative splicing affects about 95% of mammalian genes (Pan et al., 2008).

The emergence of induced pluripotent stem cells (iPSCs) provided important research tools for human disease research and drug screening (Takahashi et al., 2007; Yu et al., 2007). DS patient-derived induced pluripotent stem cells (DS hiPSCs) exhibit DS-like characteristics when they are induced to differentiate into somatic cells *in vitro* (Chou et al., 2012; Shi et al., 2012; Briggs et al., 2013) and are ideal models to study the genetic mechanisms underlying DS, as well as its associated diseases (Weick et al., 2013). Previous studies have shown that the proliferative ability of DS hiPSCs is much lower than that of normal human iPSCs (hiPSCs). This suggests that the extra chromosome 21 may also affect the biological characteristics of DS hiPSCs. However, the evidence at the molecular level is still lacking (Omori et al., 2017). In order to further explore the critical role that key genes may play in the proliferative and developmental differences between DS hiPSCs and normal hiPSCs, as well as to reveal the related molecular mechanism, we used whole genome expression profiles to analyze and screen the differentially expressed genes, and global aberrant alternative splicing events between DS hiPSCs and normal hiPSCs.

## 2. Results

### 2.1. DS hiPSCs Maintain the Pluripotent State

**Figure 1:**
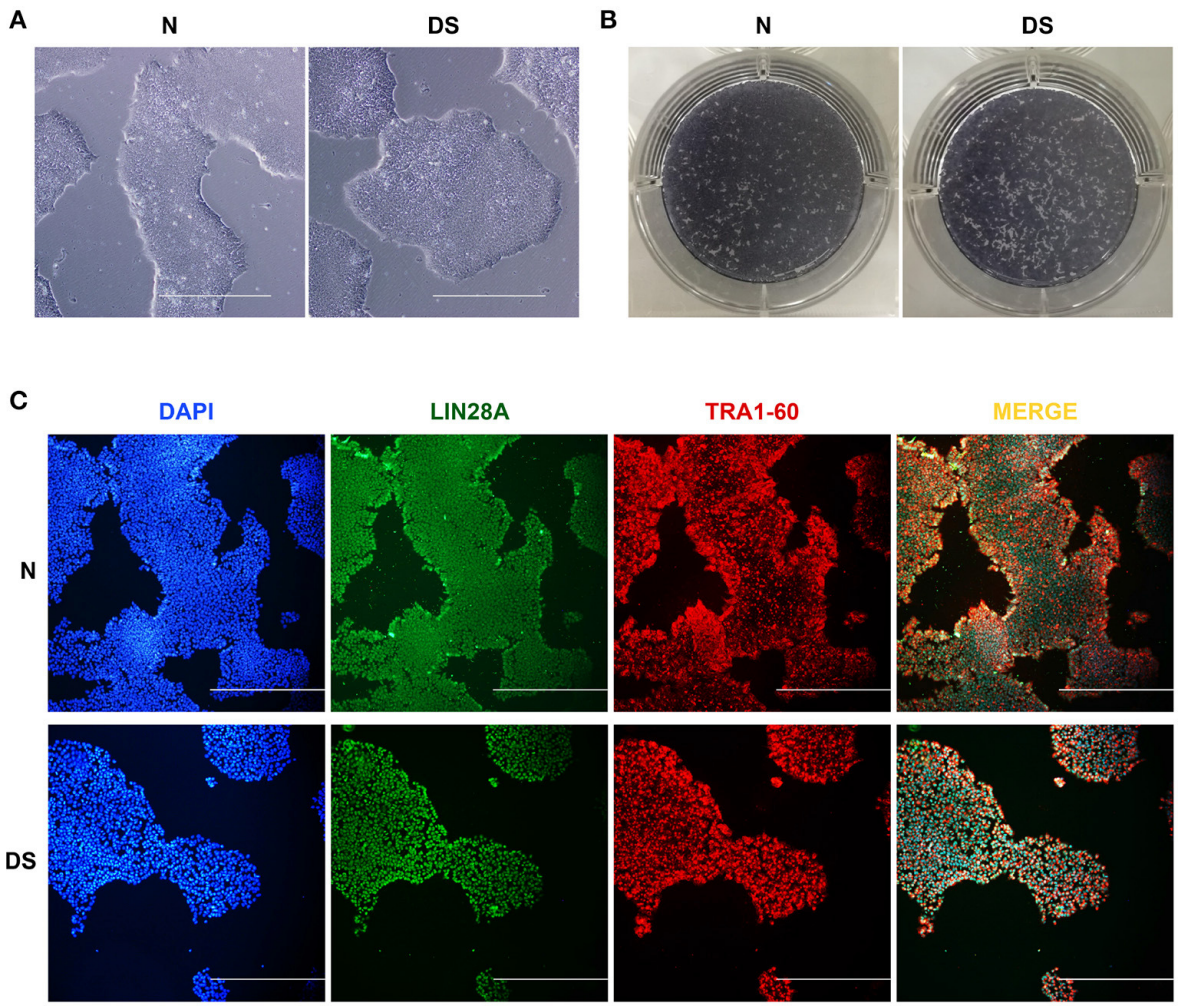
Pluripotency of hiPSCs, **(A)** Morphological observation of DS and N (Normal) hiPSCs (100×, all scale bars are 500 *μ*m), **(B)** Alkaline phosphatase staining of DS and N (Normal) hiPSCs, **(C)** Immunofluorescence images showing DAPI (blue), LIN28A (green), and TRA1-60 (red) expression in DS and N (Normal) hiPSCs (100×, all scale bars are 500 *μ*m).

To delve into whether the extra chromosome 21 affects the maintenance of pluripotency of DS hiPSCs, we primarily checked the cell morphology of DS hiPSCs and normal hiPSCs. The results showed that the DS hiPSCs exhibited pluripotent stem cells' typical characteristics with a large nucleus and compact clone (Figure 1A). A strong positive expression for alkaline phosphatase staining (Figure 1B) and the results of cell immunofluorescence assays (Figure 1C) showed no significant difference in cell morphology and surface marker expression between DS hiPSCs and normal hiPSCs. Overall, these results indicated that the maintenance of pluripotency of DS hiPSCs was similar to that of normal hiPSCs and was not significantly affected by the redundant chromosome 21.

### 2.2. Differentially Expressed Genes of DS and Healthy hiPSCs

**Figure 2:**
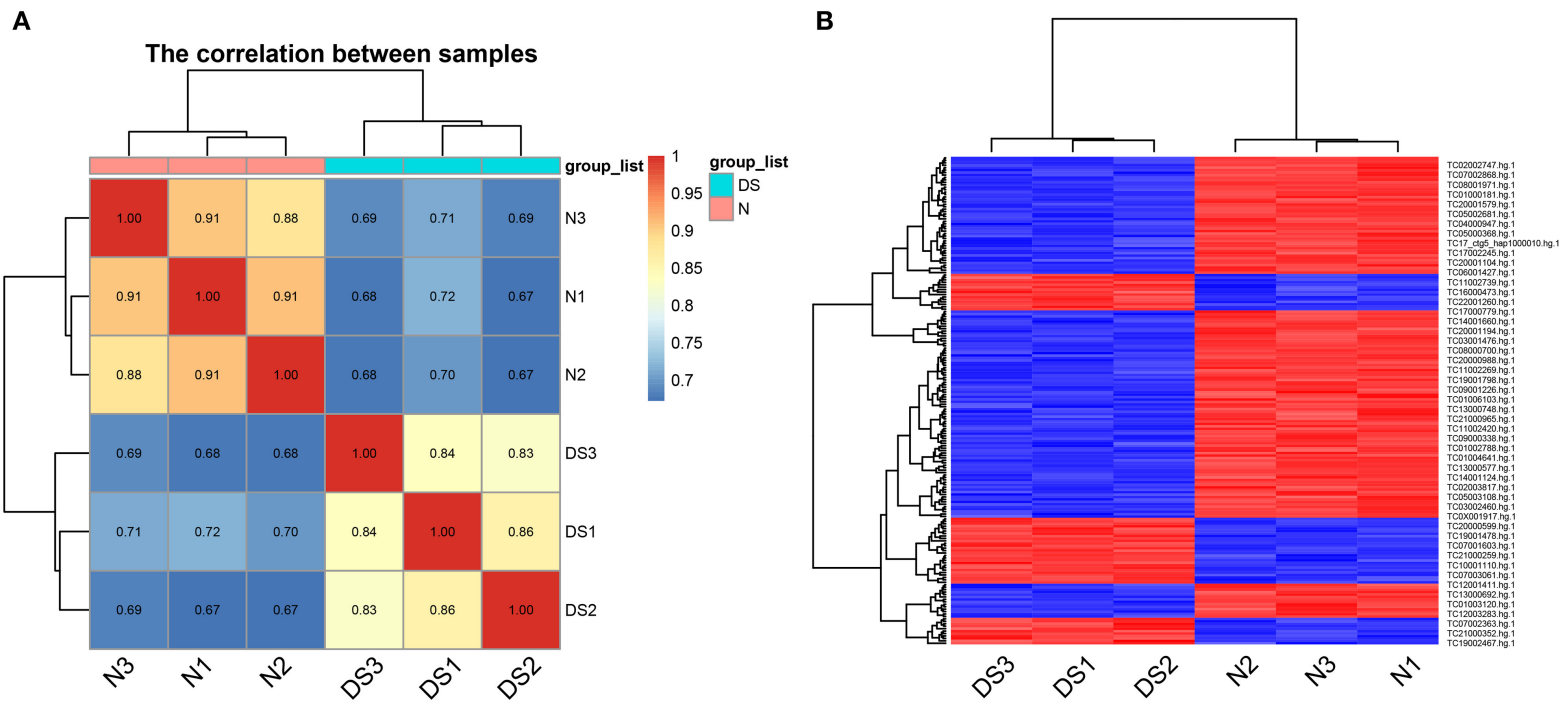
Quality control for the HTA 2.0 data, **(A)** the correlation matrix heatmap of samples, **(B)** the heatmap of differential expression probe sets.

As it is known that trisomy 21 causes alterations to both stem and precursor cells (Liu et al., 2015), it is also possible that the alteration of the proliferative ability of DS hiPSCs is caused by the differences in expression of genes and the aberrant AS events. To determine if this is the case, we used the GeneChip Human Transcriptome Array 2.0 (HTA 2.0) for the global gene analysis with hiPSCs from DS and healthy individuals. We examined differentially expressed genes (DEGs) in these groups focusing on specific transcripts with AS events. The quality control analyses of the HTA 2.0 data highlight the correct segregation of samples from each cell line (Figure 2).

**Figure 3:**
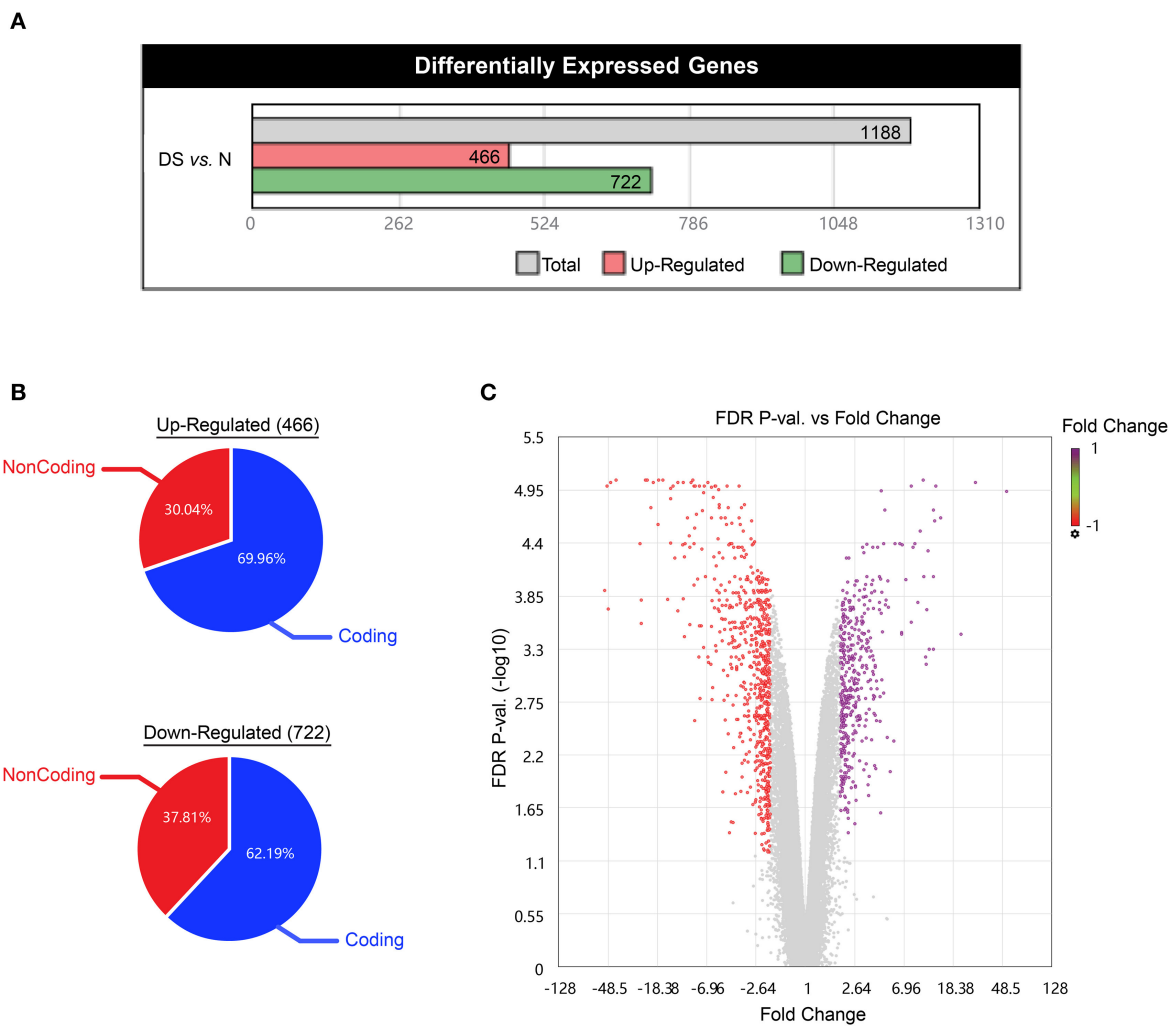
Analysis of global gene expression profile, **(A)** Differentially expressed genes (DS vs. Normal iPSCs, red and green represent up-regulated and down-regulated genes, respectively), the expression analysis settings: Gene-Level Fold Change < −2 or > 2, Gene-Level *P*-Value < 0.01, **(B)** proportion of Coding and Non-Coding in the Up-Regulated and Down-Regulated genes, Filter criteria: Fold Change: > 2 or < −2, *P*-value: < 0.01, **(C)** Volcano map of differentially expressed genes (DS vs. Normal iPSCs, red and violet represent up-regulated and down-regulated genes, respectively).

A more detailed analysis of HTA 2.0 datasets revealed, the total differentially expressed genes are 466 up-regulated and 722 down-regulated genes (in total 1,188 significantly differentially expressed genes) in DS hiPSCs, compared with normal hiPSCs (Figure 3). By summarizing the distribution of differentially expressed genes on each chromosome and the proportion of coding genes in the chromosome, we found that the proportion of up-regulated genes on chromosome 21 (5.20%) was significantly higher than that on the other chromosomes (0–1.79%), which showed a gene dosage effect of the genes on chromosome 21. This result is consistent with previous studies of DS somatic cells that demonstrated that the redundant chromosome 21 leads to gene dosage effects (Moldrich et al., 2007; Nawa et al., 2019).

Tables 1 and 2 list the top 10 up-regulated and down-regulated genes in DS hiPSCs sorted by the *P*-value, respectively. The up-regulated *H1-6* is a member of the histone H1 family. The linker histone, H1, interacts with linker DNA between nucleosomes and functions in chromatin compaction into higher-order structures. The down-regulated *H3C11* and *H4C13* are members of the histone H3 and H4 families, respectively, which are essential nuclear proteins responsible for the nucleosome structure of the chromosomal fiber in eukaryotes. The up- and down-regulation of these genes may affect the compaction of chromatin and the higher-order structures of chromosomes in DS hiPSCs, leading to the instability of chromosomes and alternative modifications of histones resulting in the aberrant regulation of gene expression. *DYRK1A*, a gene coding for a kinase known to regulate splicing factors that maps to chromosome 21 (Qian et al., 2011), was identified as an up-regulated gene in DS hiPSCs. The aberrant expression of this regulator of splicing factors may lead to splicing changes in the trisomic cells.

**Table 1:** Up-regulated genes in DS hiPSCs (Sort by *P*-value, and only the top 10 were listed).

	**logFC**	**AveExpr**	***t***	***P*-value**	**Adj. *P*-value**	**B**
*LINC02864*	2.274	4.758	36.646	5.880E-09	1.730E-05	10.570
*MT1G*	2.166	7.572	14.490	2.690E-06	6.171E-04	5.561
*MT2P1*	1.865	9.086	31.572	1.580E-08	3.290E-05	9.944
*DDR2*	1.808	5.362	29.147	2.690E-08	5.040E-05	9.575
*ODC1*	1.681	8.050	24.326	8.930E-08	1.054E-04	8.661
*H1-6*	1.647	6.121	14.977	2.170E-06	5.613E-04	5.775
*RWDD2B*	1.643	4.647	36.150	6.430E-09	1.750E-05	10.516
*ARRDC3*	1.627	5.878	35.191	7.690E-09	1.940E-05	10.408
*GTF2H2B*	1.578	5.973	12.428	7.250E-06	1.025E-03	4.557
*RNU5A-8P*	1.539	7.621	17.312	8.400E-07	3.361E-04	6.689

**Table 2:** Down-regulated genes in DS hiPSCs (Sort by *P*-value, and only the top 10 were listed).

	**logFC**	**AveExpr**	***t***	***P*-value**	**Adj. *P*-value**	**B**
*DNAJC15*	−2.474	5.292	−48.004	9.730E-10	1.090E-05	11.492
*LINC02335*	−2.408	4.742	−40.941	2.810E-09	1.390E-05	10.982
*LINC02334*	−2.316	4.670	−28.118	3.420E-08	5.490E-05	9.402
*TYW3*	−2.309	5.466	−40.264	3.140E-09	1.390E-05	10.923
*TRPC4*	−2.233	5.239	−46.314	1.240E-09	1.090E-05	11.385
*H3C11*	−2.116	9.460	−47.976	9.760E-10	1.090E-05	11.490
*GPR50*	−2.107	6.375	−22.569	1.470E-07	1.328E-04	8.251
*CRYZ*	−2.084	5.146	−43.538	1.860E-09	1.320E-05	11.190
*H4C13*	−2.024	8.563	−13.833	3.630E-06	7.249E-04	5.260
*LINC00458*	−1.869	5.330	−18.276	5.890E-07	2.738E-04	7.021

### 2.3. Differences of AS Between DS and Healthy hiPSCs

**Figure 4:**
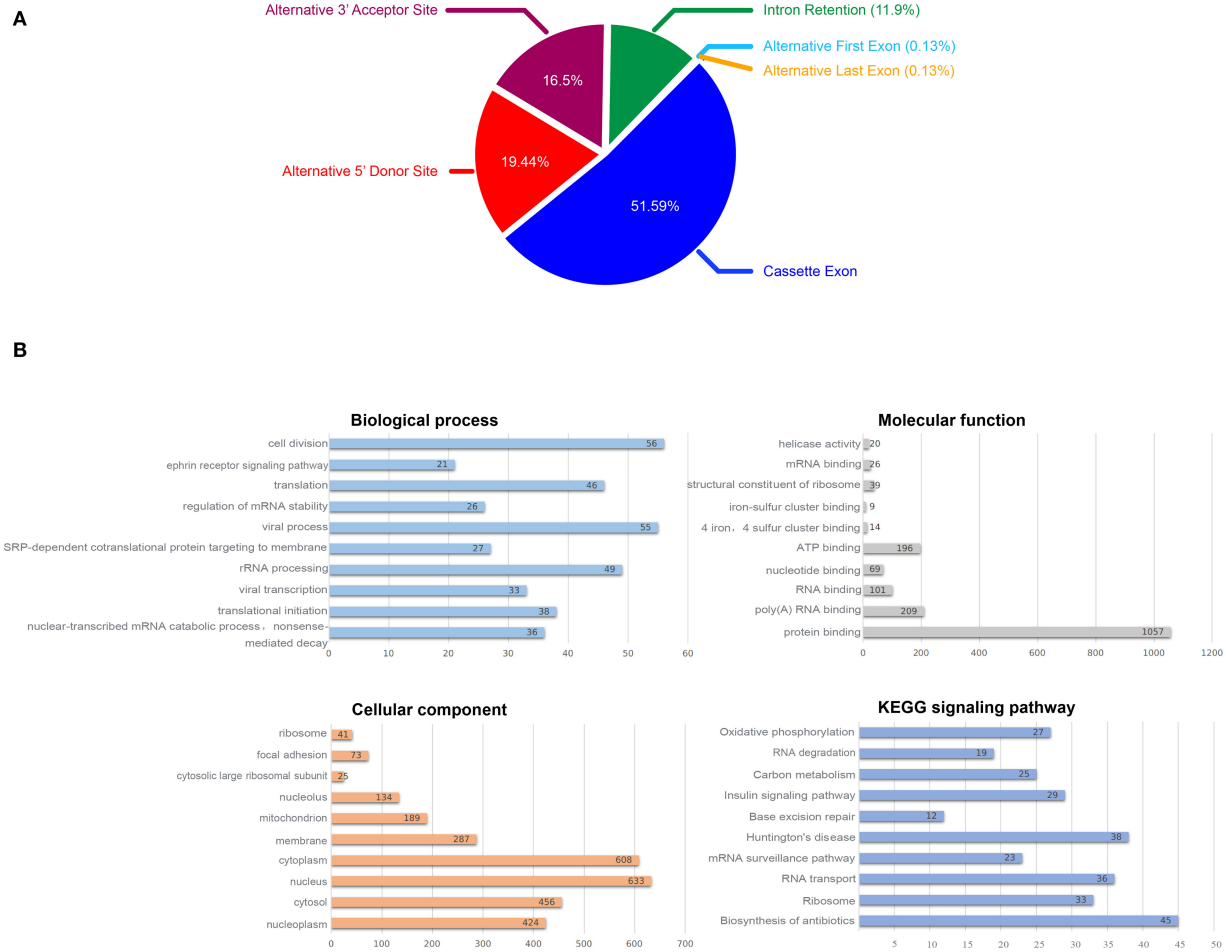
Alternative splicing analysis, **(A)** Proportion of AS events, **(B)** Biological function enrichment analysis of the identified 1,862 annotated genes with splicing changes.

We tested multiple splicing algorithms to identify differences in splicing between DS and normal hiPSCs and chose EventPointer (see Methods). Using this algorithm with the filter criteria: Exon Splicing Index > 2 or < −2 and Exon *P*-value < 0.01, we identified 1,862 annotated genes with splicing changes when comparing DS with normal hiPSCs, half of which are the Cassette Exon events and more than one third are Alternative 5′ Donor Site events and the Alternative 3′ Acceptor Site (Figure 4A).

To explore the effect of splicing changes on the DS hiPSCs, we performed the gene ontology (GO) and KEGG signaling pathway analysis on all selected genes with alternative splicing events (Figure 4B). The results showed that 1,593 genes (87.8% of 1,862 annotated genes with splicing changes) are enriched in 188 biological processes, while 1,675 genes (92.3%) were enriched in 84 cellular components. We observed enrichment for 1,601 genes (88.2%) in 75 molecular functions, as well as 707 genes (39%) in 49 KEGG signaling pathways. The top 10 enrichment items are listed in Figure 4B. Fifty-six genes with splicing changes are enriched in the cell division process; this might be the driving force behind the phenomenon that the proliferative ability of DS hiPSCs and normal hiPSCs is different.

**Figure 5:**
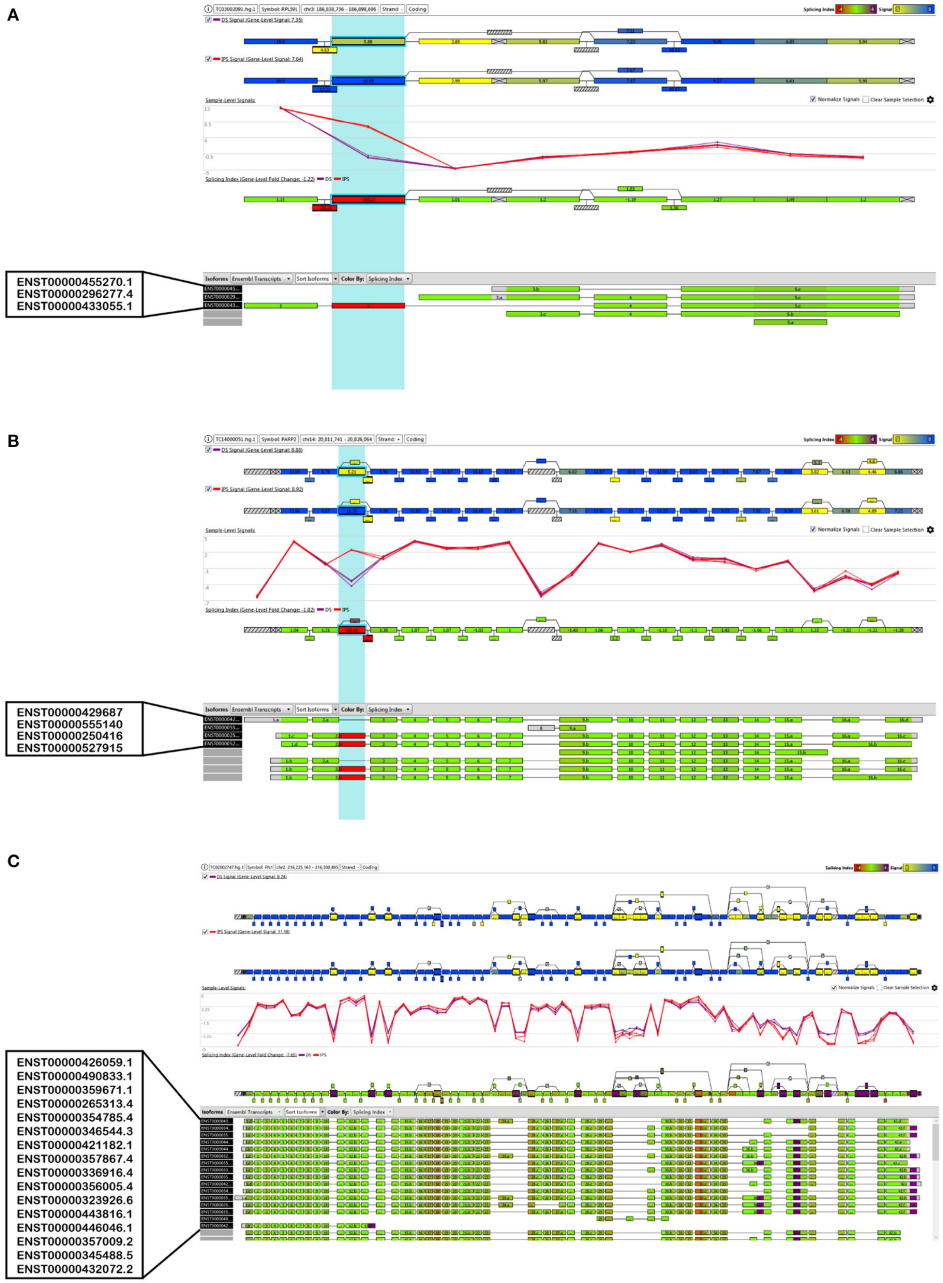
Visualization of the alternative splice events of three selected genes, **(A)** *RPL39L*, **(B)** *PARP2* and **(C)** *FN1*.

Figure 5 demonstrates the visualization of three genes as examples of splicing changes, namely the gene *RPL39L* (Ribosomal Protein L39 Like, OMIM: 607547), *PARP2* (Poly(ADP-Ribose) Polymerase 2, OMIM: 607725), and *FN1* (Fibronectin 1, OMIM: 135600).

### 2.4. Differentially Expressed Genes of DS and Healthy hiPSCs Enriched in the Biological Processes Involved in Cell Growth

To explore the effect of differentially expressed genes on the DS hiPSCs, we further performed GO functional enrichment and KEGG signaling pathway analysis of 466 up-regulated and 722 down-regulated genes, respectively. The results show that 466 up-regulated genes are enriched in 64 biological processes, 34 cellular components, 18 molecular functions, and 10 KEGG signaling pathways. The 722 down-regulated genes are enriched in 50 biological processes, 27 cellular components, 16 molecular functions, and 8 KEGG signaling pathways. The top 10 enrichment items in up- and down-regulated genes are listed in Tables 3 and 4, respectively. Eight up-regulated genes were significantly enriched in the negative regulation of growth, which may be related to somatic cells' lower proliferative ability and stem cells from patients with DS (Kimura et al., 2005). Overexpression of genes related to negative growth regulation may inhibit cell proliferation, suggesting that the proliferation of DS hiPSCs may also be impaired. Nine up-regulated genes are significantly enriched in proteins targeting the mitochondria, which may affect ATP synthesis by damaging mitochondrial function, resulting in the inhibition of select cell functions (Valenti et al., 2018). Fifteen up-regulated genes were significantly enriched in nucleosome assembly, implying that the redundant chromosome 21 may increase chromosome synthesis and assembly stress. Twenty-seven down-regulated genes were significantly enriched in cell adhesion. The impaired cell adhesion ability affects cell growth and neural cell migration during embryonic development in DS patients (Huo et al., 2018). In nervous system development there were 17 down-regulated genes that were enriched, suggesting that the molecular regulation might be abnormal before the differentiation of DS neurocytes (Liu et al., 2015). Moreover, in focal adhesion there were 16 down-regulated genes that were significantly enriched. This is consistent with the enrichment in cell adhesion of the GO analysis. Another 16 down-regulated genes were enriched in the PI3K Akt signaling pathway. The hyperactivity of this pathway promotes carcinogenesis (Yang et al., 2019). The silencing of this signaling pathway in DS hiPSCs may be the critical cause of the low incidence of solid tumors in DS patients. The disorder of the whole-genome expression profile indicates that other biological characteristics of DS hiPSCs, such as proliferation and cell adhesion, are also affected.

**Table 3:** GO and KEGG analysis of global up-regulated genes (Sort by *P*-value, and only the top ten GO items/pathways were listed in the table if there were more than 10).

**Category**	**Pathway ID**	**Pathway description**	**Count**	***P*-value**
GOTERM_BP	GO:0045926	Negative regulation of growth	8	7.25E-07
GOTERM_BP	GO:0071294	Cellular response to zinc ion	8	7.25E-07
GOTERM_BP	GO:0006626	Protein targeting mitochondrion	9	5.28E-06
GOTERM_BP	GO:0071276	Cellular response to cadmium ion	7	6.26E-06
GOTERM_BP	GO:0006334	Nucleosome assembly	15	1.19E-05
GOTERM_BP	GO:0006418	tRNA aminoacylation for protein translation	9	1.91E-05
GOTERM_BP	GO:0002381	Immunoglobulin production involved in immunoglobulin mediated immune response	4	0.000249
GOTERM_BP	GO:0042594	Response to starvation	7	0.000521
GOTERM_BP	GO:0002455	Humoral immune response mediated by circulating immunoglobulin	4	0.000835
GOTERM_BP	GO:0007059	Chromosome segregation	9	0.000889
GOTERM_CC	GO:0005829	Cytosol	150	6.53E-09
GOTERM_CC	GO:0005654	Nucleoplasm	129	3.20E-08
GOTERM_CC	GO:0005737	Cytoplasm	207	1.31E-07
GOTERM_CC	GO:0005739	Mitochondrion	68	6.35E-06
GOTERM_CC	GO:0000786	Nucleosome	13	1.70E-05
GOTERM_CC	GO:0070062	Extracellular exosome	116	5.45E-05
GOTERM_CC	GO:0005634	Nucleus	197	0.000125
GOTERM_CC	GO:0005813	Centrosome	26	0.000713
GOTERM_CC	GO:0030658	Transport vesicle membrane	7	0.000721
GOTERM_CC	GO:0048471	Perinuclear region of cytoplasm	33	0.001252
GOTERM_MF	GO:0005515	Protein binding	320	1.98E-07
GOTERM_MF	GO:0042803	Protein homodimerization activity	39	0.000682
GOTERM_MF	GO:0031492	Nucleosomal DNA binding	7	0.002361
GOTERM_MF	GO:0042802	Identical protein binding	36	0.006196
GOTERM_MF	GO:0098641	Cadherin binding involved in cell-cell adhesion	18	0.006583
GOTERM_MF	GO:0046982	Protein heterodimerization activity	25	0.006996
GOTERM_MF	GO:0042393	Histone binding	10	0.01105
GOTERM_MF	GO:0004364	Glutathione transferase activity	5	0.019906
GOTERM_MF	GO:0000175	3′-5′-exoribonuclease activity	4	0.020776
GOTERM_MF	GO:0031267	Small GTPase binding	4	0.020776
KEGG_PATHWAY	hsa04978	Mineral absorption	10	1.66E-05
KEGG_PATHWAY	hsa00670	One carbon pool by folate	6	0.00051
KEGG_PATHWAY	hsa05322	Systemic lupus erythematosus	14	0.00079
KEGG_PATHWAY	hsa00970	Aminoacyl-tRNA biosynthesis	8	0.007952
KEGG_PATHWAY	hsa01230	Biosynthesis of amino acids	8	0.01261
KEGG_PATHWAY	hsa01130	Biosynthesis of antibiotics	15	0.016534
KEGG_PATHWAY	hsa03022	Basal transcription factors	6	0.01975
KEGG_PATHWAY	hsa04115	p53 signaling pathway	7	0.029229
KEGG_PATHWAY	hsa04144	Endocytosis	15	0.042538
KEGG_PATHWAY	hsa00260	Glycine, serine, and threonine metabolism	5	0.046341

**Table 4:** GO and KEGG analysis of global down-regulated genes (Sort by *P*-value, and only the top ten GO items/pathways were listed in the table if there were more than 10).

**Category**	**Pathway ID**	**Pathway description**	**Count**	***P*-value**
GOTERM_BP	GO:0007155	Cell adhesion	27	4.14E-05
GOTERM_BP	GO:0030198	Extracellular matrix organization	14	0.000846
GOTERM_BP	GO:0006865	Amino acid transport	6	0.001333
GOTERM_BP	GO:0007156	Homophilic cell adhesion via plasma membrane adhesion molecules	12	0.001412
GOTERM_BP	GO:0007399	Nervous system development	17	0.001454
GOTERM_BP	GO:0030168	Platelet activation	10	0.001691
GOTERM_BP	GO:0042493	Response to drug	17	0.002602
GOTERM_BP	GO:0001666	Response to hypoxia	12	0.002759
GOTERM_BP	GO:0032355	Response to estradiol	8	0.005937
GOTERM_BP	GO:0019827	Stem cell population maintenance	6	0.006509
GOTERM_CC	GO:0005887	Integral component of plasma membrane	65	9.07E-08
GOTERM_CC	GO:0005886	Plasma membrane	141	1.08E-07
GOTERM_CC	GO:0031012	Extracellular matrix	21	1.60E-05
GOTERM_CC	GO:0005925	Focal adhesion	24	3.47E-05
GOTERM_CC	GO:0005578	Proteinaceous extracellular matrix	18	0.000148
GOTERM_CC	GO:0009986	Cell surface	27	0.000309
GOTERM_CC	GO:0030424	Axon	14	0.001796
GOTERM_CC	GO:0005604	Basement membrane	8	0.002139
GOTERM_CC	GO:0005615	Extracellular space	48	0.002263
GOTERM_CC	GO:0016323	Basolateral plasma membrane	12	0.002845
GOTERM_MF	GO:0005509	Calcium ion binding	40	1.16E-06
GOTERM_MF	GO:0004714	Transmembrane receptor protein tyrosine kinase activity	8	2.62E-05
GOTERM_MF	GO:0005178	Integrin binding	11	0.00019
GOTERM_MF	GO:0015171	Amino acid transmembrane transporter activity	7	0.000795
GOTERM_MF	GO:0004716	Receptor signaling protein tyrosine kinase activity	4	0.00139
GOTERM_MF	GO:0005201	Extracellular matrix structural constituent	7	0.004977
GOTERM_MF	GO:0015293	Symporter activity	6	0.006927
GOTERM_MF	GO:0043395	Heparan sulfate proteoglycan binding	4	0.008213
GOTERM_MF	GO:0043548	Phosphatidylinositol 3-kinase binding	4	0.009585
GOTERM_MF	GO:0001948	Glycoprotein binding	6	0.018656
KEGG_PATHWAY	hsa04510	Focal adhesion	16	0.000132
KEGG_PATHWAY	hsa04974	Protein digestion and absorption	8	0.005345
KEGG_PATHWAY	hsa04066	HIF-1 signaling pathway	8	0.008544
KEGG_PATHWAY	hsa05205	Proteoglycans in cancer	12	0.009214
KEGG_PATHWAY	hsa04512	ECM-receptor interaction	7	0.018733
KEGG_PATHWAY	hsa04151	PI3K-Akt signaling pathway	16	0.020078
KEGG_PATHWAY	hsa04022	cGMP-PKG signaling pathway	9	0.03806
KEGG_PATHWAY	hsa05146	Amoebiasis	7	0.043644

### 2.5. Experimental Verification of the DEG Analysis Results

**Figure 6:**
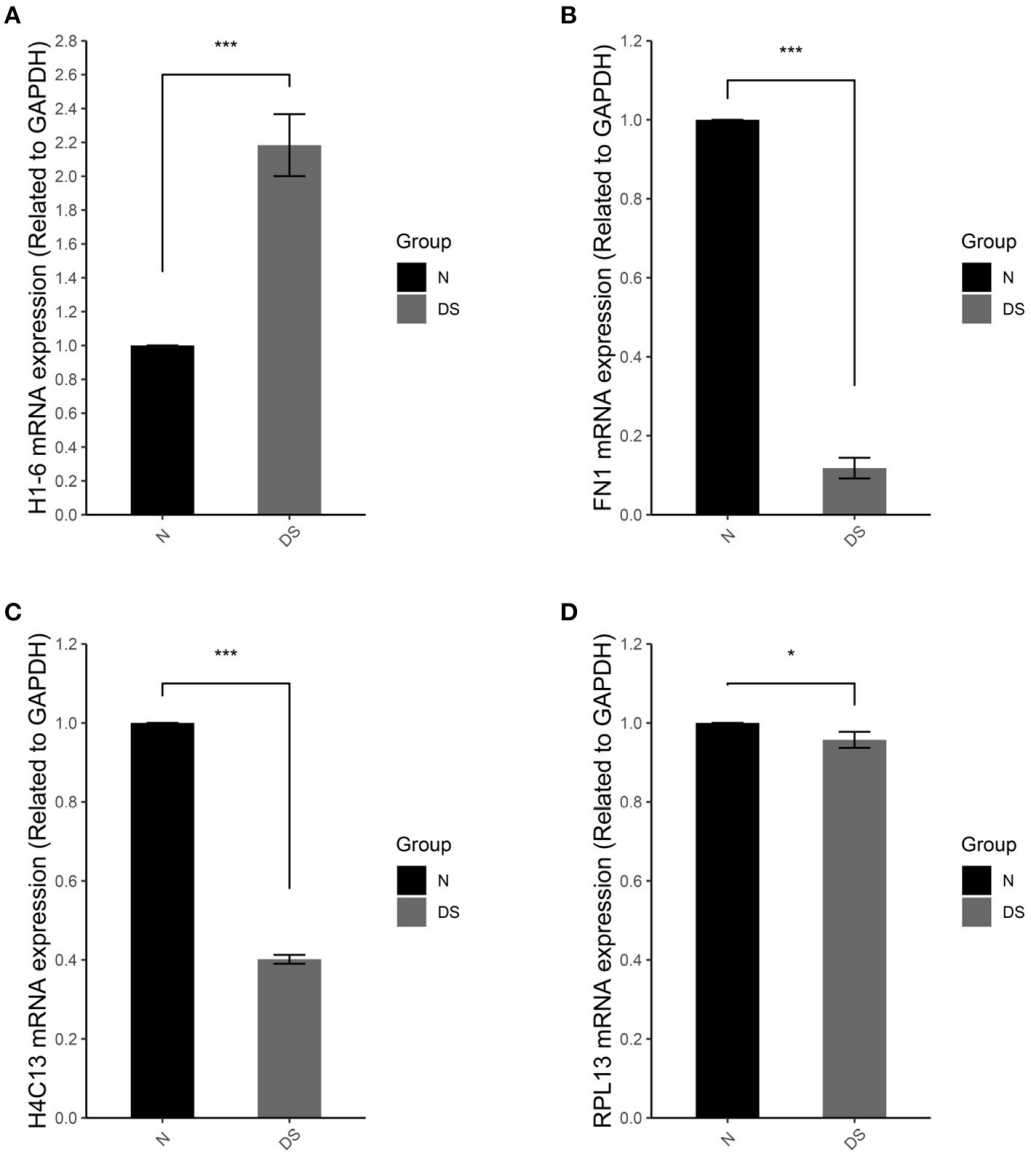
qRT-PCR of **(A)** *H1-6*, **(B)** *FN1*, **(C)** *H4C13*, and **(D)** *RPL13* in normal iPSCs (N) and DS hiPSCs (DS). Data are represented as mean ± s.e.m. * stand for *P* < 0.05 and *** for *P* < 0.001 by one-way followed with Tukeys test from *n* = 3.

To test the reliability of gene expression microarray results, several genes were selected and were validated by real time qPCR. The relative quantifications of the expression of these genes in DS hiPSCs and normal hiPSCs are represented in Figure 6.

In compare with normal hiPSCs, DS hiPSCs exhibited a 2.1-fold increase of *H1-6* (Figure 6A), a linker histone, which interacts with DNA between nucleosomes and plays an important role in the compaction of chromatin into higher order structures. In DS hiPSCs, the expressions of *FN1* (Figure 6B) and *H4C13* (Figure 6C) were reduced 4- and 2.5-fold, respectively. The expression of the gene *RPL13* (Figure 6D) was not significant changed in DS hiPSCs in comparison with the normal hiPSCs. All of these results are consistent with the DEG analysis of the microarray dataset.

## 3. Discussion

Although RNA-seq, a preferred platform for studying differentially expressed genes and alternative splicing nowadays, has some inherent advantages in comparison with microarrays, such as the ability to identify novel exons and splice junctions in an unbiased manner, the HTA 2.0 platform can nevertheless detect more weakly expressed events, which are missed in the RNA-seq results (Fumagalli et al., 2014; Wang et al., 2014; Romero et al., 2018). Furthermore, the modern microarrays, HTA 2.0, can still outperform RNA-seq for the analysis of gene expression in terms of cost, reproducibility and time as well as memory resources for treating data (Nazarov et al., 2017; Romero et al., 2018). For these reasons, we have chosen the HAT 2.0 platform as the major research approach for the AS studies. The reliability of gene expression microarray results was tested by the relative quantification of the expression of several selected genes. All of these genes showed the same change direction as they were exhibited in the DEG analysis of the microarray dataset (Figure 6).

The triploidy of chromosome 21 is the proximal cause of DS. Multi-omics studies of DS somatic cells revealed that the transcriptome and epigenetic modification disorders, leads to the disequilibrium of disease-related gene expression as a possible underlying mechanism contributing to the comorbidities associated with DS (Letourneau et al., 2014; Waugh et al., 2019). Therefore, the studies on DS's molecular regulatory mechanism can provide novel insight that could improve the clinical management of these patients. In this study, we examined differentially expressed genes (DEGs) in hiPSCs derived from DS and healthy individuals and focused on specific transcripts with AS. 1,188 differentially expressed genes and 1,862 annotated genes with splicing changes were analyzed by GO function enrichment and KEGG signaling pathway analysis, in order to dissect out the cause of the difference between DS and normal hiPSCs.

To more accurately analyze the possible dosage effects of chromosome 21 genes on DS hiPSCs, those genes determined to be up-regulated were analyzed. The biological functions and KEGG signaling pathways of the up-regulated genes on chromosome 21 suggested that dysfunctional neurodevelopment and metabolisms must have occurred in the early embryonic development of DS patients. However, the gene dosage effect can not fully explain the pathogenesis of the various comorbidities of DS patients. Bioinformatics analysis of differentially expressed genes in the whole-genome showed that the up-regulated genes were primarily involved in cell growth regulation, nucleosome assembly, and chromosome separation, suggesting that the formation and separation blockage of chromosome 21 might be the main reason for the abnormal cell cycle. KEGG signaling pathway analysis showed that the down-regulated genes are enriched in focal adhesion and extracellular matrix (ECM) receptor interaction, indicating that the migration and adhesion of DS hiPSCs may be impaired, and migration and colonization of both nervous and reproductive systems occurred during early embryonic development (Huo et al., 2018). In addition, abnormal cell adhesion was also found in the bioinformatics analysis of other DS hiPSCs (Hibaoui et al., 2014; Gonzales et al., 2018). These results are consistent with the other studies, in which the RNA-seq are used as the major research platform (Hibaoui et al., 2014; Gonzales et al., 2018; Perepitchka et al., 2020).

Key genes identified from the HTA 2.0 dataset analysis are histone coding genes involved in nucleosome formation and gene expression regulation. It appeared that alternation of histone-related genes, such as mutations in histone-modified genes, can lead to disorders related to DS, such as congenital heart disease (Zaidi et al., 2013). Some of the key genes identified in this study are involved in disorders of neurodevelopment and neurogenesis. For example, *H3C2* plays an essential role in brain development in early embryonic development (Ren and van Nocker, 2016), and *H2BC12* is involved in Alzheimer's disease (Pedrero-Prieto et al., 2019). *H1-2*, on the other hand, plays a critical role in the stabilization of chromatin and is involved in cell cycles, apoptosis induced by DNA damage, and the stabilization of autophagy protein and fibrin (Konishi et al., 2003; Sancho et al., 2008; Roque et al., 2015; Wang et al., 2017). The up-regulated *H1-6* and down-regulated *H3C11* and *H4C13* are also essential for the stabilization and structure of chromatin and chromosomes. The alteration of the expression of these genes could also lead to variations of chromatin states and chromosome structure. These genes are worthy of further analysis to determine the reasonable correlations to pathological knowledge.

We observed trisomy-dependent splicing changes in the DS hiPSCs. Our identification of splicing changes resonance with the alternative splicing events in a selected set of genes in fetal DS tissue that has been previously reported (Toiber et al., 2010). AS in DS endothelial progenitor cells has also been analyzed via RNA-seq, although no confirmatory studies have yet been performed (Costa et al., 2011). The large majority of alternative splicing events we identified occur in genes located outside of chromosome 21, implying that these AS differences were not directly affected by gene dosage. It is more likely that altered expression or altered splicing activity could cause the splicing dysregulation in DS hiPSCs. Alternatively, up-regulation of the chromosome 21 gene, such as *DYRK1A*, a kinase known to regulate splicing factors (Qian et al., 2011), could contribute to the alternative splicing events we identified. It has been reported that over-expression of *DYRK1A* in mice led to mimicry of splicing aberrations in DS (Toiber et al., 2010). The role of *DYRK1A* or the other specific splicing factors in the alternative splicing changes we identified will require additional studies. The gene dosage effect and expression levels of these genes should be normalized in the DS hiPSCs backgrounds.

## 4. Materials and Methods

### 4.1. HiPSC Culture

DS hiPSC (ATCC® ACS-1003™) and normal hiPSC (ATCC® ACS-1011™) were from the ATCC (American Type Culture Collection) cell bank. HiPSCs were cultured using Gibco's StemFlex™ medium (ThermoFisher Scientific, USA). The petri dishes were pre-treated with Matrigel, and the medium was changed daily. After culturing for 4–5 days, the iPSCs were digested, and the suspended cell clusters were collected and subcultured at a ratio of 1:6 at 37°C, 5% CO_2_ (Aalders et al., 2019).

### 4.2. Cellular Immunofluorescence Experiment

After removing the culture medium, hiPSCs were washed twice with PBS, adding blocking solution (PBS solution containing 0.6% BSA) within 30 min, a membrane breaker (PBS solution containing 0.02% Triton X-100) was added to break the cell membrane. The diluted primary antibody (1:250) [TRA1-60 primary antibody (ThermoFisher Scientific, USA) and LIN28A primary antibody (Cell Signaling, USA)] were added and incubated at 4°C overnight. The cells were washed twice with PBS the next day and then incubated with the fluorescent secondary antibody (1:500) [FITC-labeled goat anti-rabbit IgG (H + L), Cy3 labeled goat anti-mouse IgG (H + L)], respectively, at room temperature, avoiding light for 2 h. Finally, the anti-fluorescence quenching mount solution (containing DAPI) was added and incubated at room temperature for 10 min before the cells were observed under a fluorescence microscope (Weltner et al., 2018).

### 4.3. Alkaline Phosphatase Staining

According to the instructions of the BCIP/NBT alkaline phosphatase staining kit's manufacturer (Beyotime Biotechnology, Shanghai, China), the protocol involved mixing 3 mL of alkaline phosphatase staining buffer with 10 *μ*L of BCIP solution (300×) and 20 *μ*L of NBT solution (150×) to prepare the working solution. Cells in 6-well plate were washed twice with PBS, then an appropriate amount of working solution was added to a single well of the 6-well plate while avoiding light exposure for 5–30 min until the color displayed to the expected depth. The working solution was then removed, and the cells were washed twice with distilled water to stop coloring. Before the photographs were taken, the stained cells were dried at room temperature and keep away from light.

### 4.4. Extraction of Total RNA

DS hiPSC and normal hiPSC were seeded in a 6-well plate, each cultured for 3 days, and then digested to harvest about 5 × 10^5^ cells. The experiment was repeated three times. The total RNA was extracted with TRIzol™ reagent (ThermoFisher Scientific, USA) and was first detected by the Agilent 2100 bioanalyzer with the Agilent RNA 6000 Nano Kit (Agilent Technologies, Waldbronn, Germany) according to the instruction of the manufacture. Total RNAs with an RNA integrity index (RIN) >7.0 were used for subsequent experiments.

### 4.5. Whole Transcriptome Profile Detection

By using WT Amplification Kit Module 1 and WT Amplification Kit Module 2 in the GeneChip™ WT PLUS Kit (ThermoFisher Scientific, USA), 100 ng of total RNA as input was performed *in vitro* transcription (IVT) to synthesize cRNA, and then reverse transcription and purification to obtain single-stranded cDNA (sscDNA). The GeneChip™ WT end labeling kit was used to fragment and label sscDNA, which was hybridized with the human transcriptome array (HTA 2.0), and the hybridization signals were detected on a chip scanner to obtain CEL files for DS and normal hiPSCs.

### 4.6. Real Time qPCR

The SuperScript™ IV reverse transcriptase (Invitrogen™, USA) was used for the synthesis of first stand cDNA from the isolated RNA. 1 *μ*g of total RNAs were reverse transcribed according to the manufacturer's instructions. cDNA was real time polymerase chain (PCR) amplified in a LightCycler® 96 System (Roche, USA) using the FastStart Essential DNA Green Master (Roche, USA). The LightCycler® 96 SW 1.1 software was used for raw data collection and gene expression comparisons (2^−ΔΔCT^ method). The R packages “Rmisc” and “multcomp” are used for the data analysis and “ggplot2” for the visualization of the results.

### 4.7. Bioinformatic Analysis

#### 4.7.1. Screening of Differentially Expressed Genes

All data were reprocessed from raw images. Signal intensities, quality control, data normalization, and gene expression values for these samples were processed together with the RMA processing algorithm using the “oligo” package in R (Carvalho and Irizarry, 2010). The DEG analysis was performed with Fold change (FC) < −2 or > 2, *P*-value < 0.01.

#### 4.7.2. Alternative Splicing Events Analysis

The alternative splicing events analysis was performed using the “EventPointer” package in R (Romero et al., 2016) with the splicing index < −2 or > 2 and exon-Level *P*-value < 0.01.

#### 4.7.3. Gene Ontology (GO) and KEGG Signaling Pathway Analysis

GO enrichment was performed using DAVID (Huang et al., 2009a,b) (http://david.abcc.ncifcrf.gov/). A hypergeometric test with the Benjamini and Hochberg false discovery rate (FDR) was performed using the default parameters to adjust the *P*-value (Benjamini and Hochberg, 1995).

## Data Availability Statement

The datasets presented in this study can be found in online repositories. The names of the repository/repositories and accession number(s) can be found at: https://www.ncbi.nlm.nih.gov/geo/query/acc.cgi?acc=GSE168111.

## Author Contributions

YW, QM, YZe and FZ were the main contributors to designing experiments. YW, QM, and FZ interpreted results, wrote and revised the manuscript. ZL and QM performed microarray experiments and analyzed data. GY, LC and YZha performed cell culture experiments and analyzed data. FY analyzed microarray data. All authors agree to be accountable for the content of the work.

## Conflict of Interest

The authors declare that the research was conducted in the absence of any commercial or financial relationships that could be construed as a potential conflict of interest.
